# Reproducibility and reliability of SNP analysis using human cellular DNA at or near nanogram levels

**DOI:** 10.1186/1756-0500-6-515

**Published:** 2013-12-06

**Authors:** Cindy Y Okitsu, David J Van Den Berg, Michael R Lieber, Chih-Lin Hsieh

**Affiliations:** 1 Department of Urology, University of Southern California, 1441 Eastlake Ave.,Rm 5420, Los Angeles, CA 90033, USA; 2 Department of Biochemistry and Molecular Biology, University of Southern California, Los Angeles, CA 90033, USA; 3 Department of Preventive Medicine, University of Southern California, Los Angeles, CA 90033, USA; 4 Department of Pathology, Norris Cancer Center, University of Southern California, Los Angeles, CA 90033, USA

**Keywords:** Chromosome deletion, Reproducibility of SNP array analysis, Reduced DNA quantity, SNP array

## Abstract

**Background:**

Illumina SNP arrays have been routinely used for genome-wide association studies to identify potential biomarkers for various diseases. The recommended 200 ng of DNA for high-quality results is a roadblock to utilizing this assay when such quantities of DNA are not available. The goal of this study is to determine the reproducibility and reliability of the assay when reduced amounts of DNA are used for the SNP arrays.

**Findings:**

A serial 3-fold reduction of DNA from 200 ng to 0.8 ng was used for an Illumina SNP array in duplicates (200 ng, 66.7 ng, 22,2 ng, and 7.4 ng) or triplicates (2.47 ng and 0.8 ng). The reproducibility of the assay was determined by comparing allele calls (genotypes) at each locus within the duplicates or triplicates. The reliability of samples of reduced quantity was determined by comparing allele calls from samples of different quantities. As expected, the reproducibility and reliability both decrease with decreasing amounts of DNA used for the arrays. However, results of comparable quality to the 200 ng DNA recommended by Illumina can be obtained with much reduced amounts of DNA.

**Conclusion:**

Reasonably reproducible and reliable results can be obtained with quantities of DNA, as low as 0.8 ng (equivalent to 133 human cells), well below the manufacturer’s recommendation. Results of nearly equal quality to that of using 200 ng DNA can be obtained with 22.2 ng of DNA reliably, and clearly acceptable data can be obtained using 7.4 ng of DNA for Illumina SNP arrays.

## Findings

### Study design

Illumina SNP arrays have been routinely used for genome-wide association studies (GWAS) to identify potential biomarkers for various diseases. The recommended 200 ng of DNA for high quality results prohibits the application of this assay when such quantities of DNA are not available. Under conditions where only about 2000 cells (equivalent to 12 ng of DNA) were available for each SNP array analysis, we carried out a study of controls to assess the reproducibility and reliability of the results using decreasing amounts of DNA from the 200 ng recommended by Illumina. The design of this study is to include six different quantities of DNA as a serial 3-fold reduction from 200 ng to 0.8 ng for each Illumina SNP array with a total of 730,525 SNPs. Each of the four higher quantities was done in duplicate and triplicates were done for the two lowest quantities. Allele calls among the duplicate or the triplicate samples of the same DNA quantity were compared to determine the reproducibility of the assay. The reliability of allele calls from samples of reduced quantity was evaluated by comparing calls from samples of lower quantities and from samples of higher quantities. It is the goal of this study to determine 1) whether decreased quantities of DNA used reduce the reproducibility of the assay; 2) the reliability of the results when reduced amounts of DNA are used for the assay; and 3) the lowest amount of DNA that permits equally reliable results as the recommended amount for the assay.

## Methods

Genomic DNA from Nalm6, a human pre-B cell line, was purified using the proteinase K/phenol/chloroform extraction method. Two samples each with 200 ng, 66.7 ng, 22.2 ng, and 7.4 ng, and three samples each with 2.47 ng, and 0.8 ng of DNA from the same extraction of Nalm6 cells were processed and hybridized to the same lot of HumanOmniExpress SNP microarrays using the standard Illumina protocol. All current Illumina SNP arrays utilize Infinium genotyping chemistry [1].

The SNPs with no calls or discordant calls from the duplicate or the triplicate samples are identified for the analysis.

## Results

### Failure rate of the assay increases only slightly with decreasing quantity of input DNA

Genotype calls are made in Genome Studio Software (Illumina; v2011.1) with Genotyping module (v1.9.4) using predefined cluster definitions for all 3 genotypes (provided by Illumina). If a genotype datapoint resides outside the clusters that define each of the 3 genotype combinations (two homozygotes and a heterozygote) then no genotype call is made. An overall call rate is calculated from dividing the number of loci with genotype calls by the total number of loci on the array. In the Illumina Infinium genotyping assay, a >99% overall call rate is considered as high-quality data and a >95% overall call rate is commonly considered to reflect acceptable quality. The overall call rate can be affected by the inadequacy of the quantity or quality of the DNA used for the array. In the current experiments, it is expected that the overall call rate of the array would decrease with decreasing quantity of DNA used for the assay, as the DNA is of the same quality. Surprisingly, the overall call rate decreases only slightly from 99.6% to 96.3% as the quantity of DNA used for the array decreases from 200 ng to 0.8 ng (Table 1). Based on the overall call rate, as little as 7.4 ng of DNA can be used to obtain high quality data with higher than 99% call rate, and even 0.8 ng of DNA can be used to generate consistently acceptable quality data.

**Table 1:** Overall call rate of Illumina SNP arrays utilizing different quantities of starting DNA

**DNA quantity**	**Call rate**
200	0.9967
200	0.9964
66.7	0.9962
66.7	0.9961
22.2	0.9951
22.2	0.9959
7.4	0.9944
7.4	0.9916
2.47	0.9804
2.47	0.9878
2.47	0.9864
0.8	0.9658
0.8	0.9736
0.8	0.9632

### Integrity of the allelic intensity ratio remains high even with much decreased quantities of DNA used

The fluorescent dye intensities of the two alleles of a SNP are used to calculate an overall signal intensity and allele intensity ratio (theta) for plotting of data points on polar coordinates to generate allele calls and the B allele frequency (BAF). The BAF (an assay “normalized” version of theta) is often used to determine whether allelic imbalances, such as loss of allele or gene amplification, exist in the sample [2,3]. In normal diploid cells, loci with homozygous alleles are expected to have a BAF (or theta) of either 1 or 0, and loci with heterozygous alleles would have a BAF (or theta) of 0.5. Therefore, deviation of BAF (or theta) from the theoretical value would reflect the quality of data generated, and we expect theta to deviate more with decreasing quantities of DNA used. It is found that the average theta does not show a clear trend of digression from theoretical values, and the standard deviation of theta only increases slightly as the quantity of DNA decreases (Table 2). This indicates that there is a slight increase in the scattering of data points as the quantity of DNA used for the array decreases; however, the scattering is not directional toward either of the two alleles. This finding further indicates that the quality of data remains high, despite the drastically reduced quantity of DNA used.

**Table 2:** Summary of allele intensity ratio from arrays utilizing various quantities of starting DNA

**Allele call**	**AB**		**AA**		**BB**	
**DNA quantity**	**Average**	**Standard deviation**	**Average**	**Standard deviation**	**Average**	**Standard deviation**
200	0.536	0.084	0.033	0.027	0.977	0.018
200	0.530	0.085	0.036	0.028	0.975	0.019
66.7	0.529	0.095	0.034	0.027	0.973	0.019
66.7	0.530	0.093	0.033	0.027	0.974	0.019
22.2	0.526	0.097	0.033	0.028	0.971	0.021
22.2	0.526	0.093	0.037	0.028	0.970	0.021
7.4	0.535	0.101	0.039	0.031	0.972	0.022
7.4	0.545	0.098	0.041	0.033	0.971	0.023
2.47	0.514	0.111	0.039	0.034	0.964	0.026
2.47	0.528	0.110	0.039	0.033	0.968	0.024
2.47	0.528	0.109	0.039	0.033	0.967	0.024
0.8	0.538	0.120	0.044	0.036	0.966	0.026
0.8	0.547	0.117	0.047	0.036	0.967	0.025
0.8	0.545	0.114	0.046	0.036	0.965	0.026

### Analysis of failed calls

The no-call (NC) SNPs may include the intrinsically low quality assays in the array, inadequate amplification of DNA in the whole genome amplification step of the assay due to the low amount of DNA used, and randomly occurring poor hybridization. The intrinsically low quality assays most likely would lead to consistent failure of genotype calls in all or most of the arrays in the current experiment and would give rise to similar numbers of NC in each array, regardless of the quantity of DNA used. The NC resulting from inadequate amplification of DNA would lead to the increased number of NC in the arrays utilizing lower quantities of DNA. In addition, random poor hybridization can occur and lead to differences, even in the duplicate or triplicate arrays using the same quantities.

There are 399 SNPs in a region that is deleted from both copies of chromosome 9 of the Nalm-6 cell line used here. Excluding these 399 SNPs, a total of 1235 SNPs had a NC readout among the 730,126 SNPs examined in both samples of 200 ng DNA (Table 3). The number of SNPs that had a NC readout in all samples of the same DNA quantity increases markedly when 7.4 ng or less of DNA was used (Table 3). When NC occurs at a locus in two of the three samples of the same quantity, the SNP should also be considered as failing consistently since the allele call has failed more often than it has succeeded. In the triplicates of the 2.47 ng DNA sample, a total of 7640 SNPs having a NC readout in at least two of the three samples (4618 NC SNPs in two samples and 3022 NC SNPs in all three samples, Table 3). Likewise, a total of 16,974 SNPs had at least two NCs in the triplicates of the 0.8 ng DNA sample (11097 NC SNPs in two samples and 5876 NC SNPs in all three samples, Table 3). It is clear that the number of consistently failed SNPs increases very quickly when less than 7.4 ng DNA was used for the array.

**Table 3:** Summary of problem allele calls in arrays utilizing different quantities of starting DNA

**A. duplicate samples**				
**Type of problems**	**DNA quantity (ng)**			
	**200**	**66.7**	**22.2**	**7.4**
NC in both samples	1235	1313	1459	2077
NC in one sample, call in the other	1778	2212	2829	5319
Different calls in two samples	17	22	22	48
Total problem calls	3030	3547	4310	7444
Problem call %	0.42	0.49	0.59	1.02
**B. triplicate samples**				
**Type of problems**	**DNA quantity (ng)**			
	**2.47**	**0.8**		
NC in all samples	3022	5876		
NC in two samples, call in the third one	4618	11097		
NC in one sample, different calls in the other two	316	738		
NC in one sample, same calls in the other two	13304	29421		
Subtotal	18238	41256		
Calls in all three, 1 incorrect call*	199	391		
Calls in all three, 2 incorrect calls*	35	148		
Calls in all three, correctness uncertain*	6	4		
Subtotal	240	543		
Total problem calls	21500	47675		
Problem call %	3.00	6.58		

*Correctness of the call is determined by the calls in the arrays using higher quantity of DNA samples.

A total of 587 SNPs (0.08%) had consistent NC results in all 14 samples. These SNPs are not contiguous SNPs and are not likely to be sites of homozygous deletion. These consistently failed SNPs are most likely the result of the very small percentage of intrinsically poor quality assays, even though we cannot completely rule out sporadic homozygous deletion events in the cells.

### Analysis of inconsistent calls

While the rate of NC reflects the overall data quality, the lack of information at these NC SNPs would not lead to any conclusion. However, incorrect allele calls would potentially lead to misleading conclusions. Examining inconsistent calls of alleles from different arrays of the same DNA sample can reveal the reproducibility of the assay as well as how reliable the assay is when samples with suboptimal quantities of DNA are used. Therefore, it is important to know the rate of false call changes with various quantities of DNA used. There are fewer than 50 inconsistent calls when 7.4 ng or more of DNA was used for the array (0.002% to 0.007% of total SNPs), and the number of inconsistent calls increased to 543 (0.07%) when 0.8 ng of DNA was used (Table 3). The rate of discordant calls within the duplicates is consistent with the 0.1% to 0.15% reported previously using 500 ng DNA for the Illumina 1MDuo chip [4]. These false calls are not at the same SNPs across all arrays utilizing different quantities of DNA, even though few occurred in more than one array. This finding indicates that the false calls are more likely random in nature and not due to a hybridization artifact intrinsic to the specific SNP assay. The total number of inconsistent calls remains low even though it increased more than 10-fold in DNA samples of 0.8 ng compared with DNA samples of 7.4 ng. While the total number of problem calls, including NC in any samples and inconsistent calls, increases from 3,030 in DNA samples of 200 ng to 47,675 in DNA samples of 0.8 ng (Table 3), it is clear that the cause of this increase is mostly due to NCs (41,256 SNPs involve a NC read out in at least one of the three 0.8 ng samples). These findings suggest that the reproducibility within the duplicates using 7.4 ng of DNA is nearly as good as using 200 ng of DNA. Also, the reliability of the results remain high in 0.8 ng DNA samples because the rapid rise of problem calls is the consequence of failure to make an allele call and not the consequence of making an incorrect or inconsistent calls.

### Capability of detecting large deletions is not compromised by a decreased quantity of input DNA

Based on conventional karyotypic analysis, the Nalm-6 cells have a known deletion that spans several chromosomal bands of more than 8 Mb DNA in the long arm of a chromosome 5. This deletion is clearly detected in all of the arrays in the current study, and the number of SNPs with NC or conflicting calls in the region of the deletion is very small and not any higher than other parts of the genome. This would indicate that the quality of data and the capability of detecting allele loss are not compromised even at the lowest quantity of 0.8 ng of DNA used (equivalent to 133 cells).

**Figure 1:**
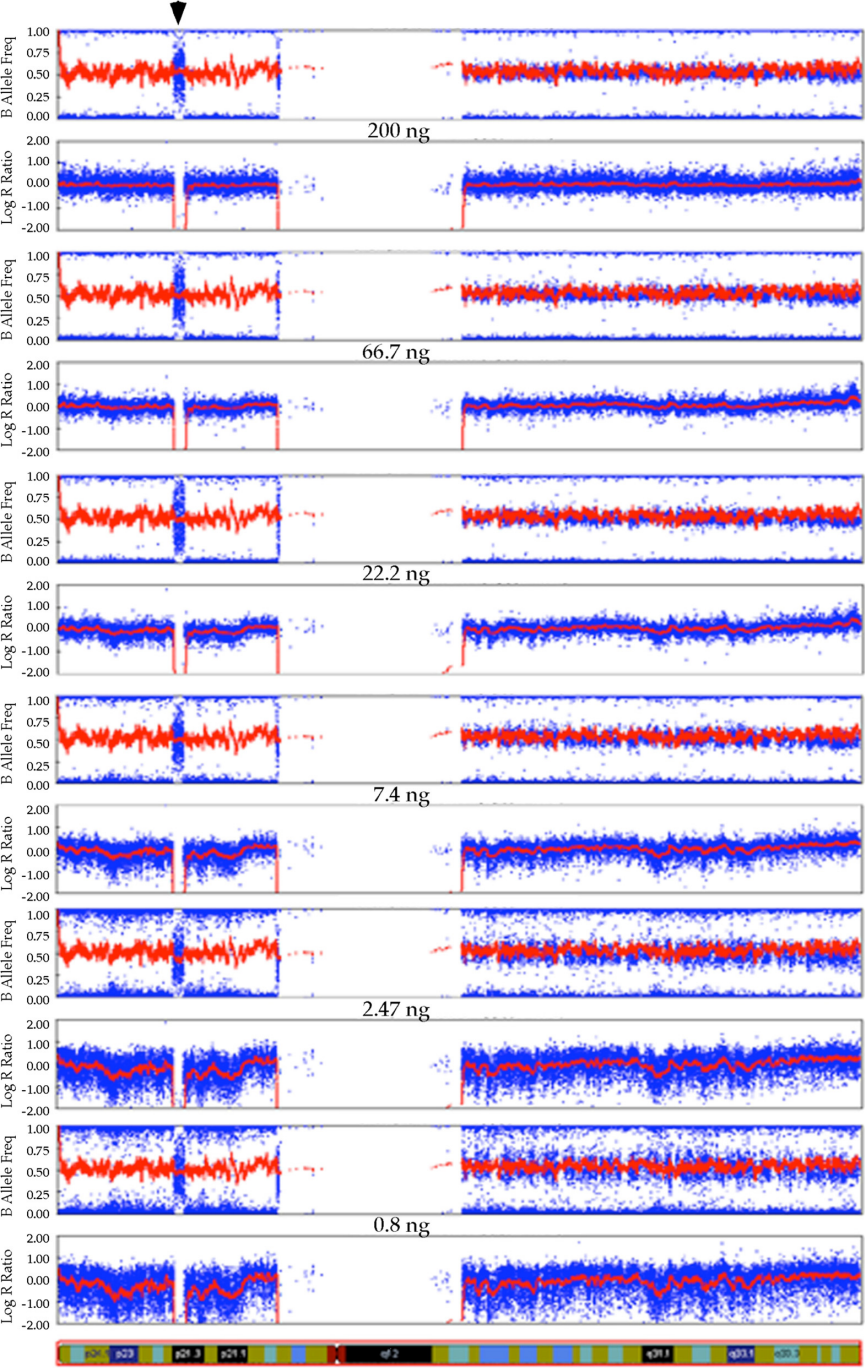
**Detection of complete homozygosity of the short arm including a small deletion in the chromosome 9.** The B allele frequency (BAF) and Log R ratio (LRR) plots of SNP calls on chromosome 9 (idiogram displayed on the bottom of the diagram) using various quantities of DNA for the array are shown. The BAF in the short arm are 1 or 0, except the small region of deletion, indicating homozygosity of the entire short arm. The deleted region is indicated with an arrow on the top of the figure. The LRR is clearly absent from 0, which would indicate two copies of the chromosome are present. It is apparent that as the DNA quantity decreases, the level of noise and scattering increases in both plots. However, the region of deletion is clearly identifiable in all arrays.

An unexpected finding is that almost all loci on the short arm of chromosome 9 had homozygous calls except a small region of 1.8 Mb with contiguous NC SNPs in all 14 arrays (Figure 1). There are two copies of apparently normal chromosome 9 based on the karyotype and the logR ratio (deviation from 0 indicates copy number change) of the arrays. This finding indicates that a gene conversion or duplication event may have led to the complete homozygosity, including a deletion of less than 2 Mb, on the short arms of the two chromosome 9. This 1.8 Mb homozygous deletion can be consistently and confidently detected even when very low quantities of DNA (0.8 ng) are used for the array. It is noteworthy that there are sporadic allele calls in this region of homozygous deletion that appear to be random in nature, and these are much more frequently called as heterozygotes than homozygotes.

## Conclusions

The goal of this study was to explore the feasibility, reproducibility, and reliability of SNP analysis using much less input DNA than the recommended 200 ng for an Illumina SNP array. The findings in our study will facilitate studies that only very small quantity of DNA is available for analysis. In general, the quality of data from Illumina SNP array remains high despite the fact that the quantity of DNA used was reduced to as low as 0.8 ng from the 200 ng recommended by the manufacturer. The overall failed calls increased to just above 3.7% for the lowest quantity of 0.8 ng DNA from just over 0.3% for the 200 ng DNA. Although the variation in the intensity ratio of the allele detection increases (more scatter) with lower quantities of DNA used, the overall reproducibility and reliability of the data do not appear to be compromised. In addition to some SNPs with intrinsic assay problems and some randomly occurring failed allele calls, a small number of incorrect calls occur at random loci, even when the recommended 200 ng of DNA is used. We conclude that a nearly equal quality of data can be obtained using 22.2 ng of DNA as using 200 ng of DNA, and reproducibility as well as reliability of the data is clearly acceptable when 7.4 ng or more of DNA is used for Illumina SNP arrays. A small deletion of 1.8 Mb was detected consistently in all 14 arrays indicating the reliability of using suboptimal quantity of DNA, as low as 0.8 ng, in detecting small deletion. However, regardless of the quantity of DNA used, caution should still be exercised and confirmatory studies should certainly be done using independent assays and other types of assays when important associations to the SNP are believed to occur.

## Competing interests

The authors declare that they have no competing interests.

## Authors’ contributions

CYO analyzed the SNP data, DJVDB carried out the array hybridization work, All authors participated in experimental design, data analysis strategies, and manuscript preparation. All authors read and approved the final manuscript.
